# Changes in Etiologies of Hospitalized Patients with Liver Cirrhosis in Beijing 302 Hospital from 2002 to 2013

**DOI:** 10.1155/2017/5605981

**Published:** 2017-11-19

**Authors:** Binxia Chang, Baosen Li, Ying Sun, Guangju Teng, Ang Huang, Jin Li, Zhengsheng Zou

**Affiliations:** ^1^Center for Diagnosis and Treatment of Non-Infectious Liver Disease, Beijing 302 Hospital, Beijing 100039, China; ^2^Department of Medical Administration, Beijing 302 Hospital, Beijing 100039, China

## Abstract

**Background:**

Over the last 20 years, the prevalence of hepatitis B virus (HBV) infection in China has decreased gradually due to the application of a national HBV vaccination program. In contrast, the prevalence of alcoholic liver disease (ALD), nonalcoholic fatty liver disease, autoimmune liver disease, and drug-induced liver injury has markedly increased.

**Methods:**

We conducted a retrospective review of 82,562 hospitalized patients diagnosed with liver cirrhosis in Beijing 302 Hospital from 2002 to 2013.

**Results:**

The top four etiologies of cirrhosis were HBV, HCV, ALD, and autoimmune liver disease. The percentage of HBV cirrhosis decreased from 81.53% in 2002 to 66.0% in 2013, whereas the frequency of alcoholic cirrhosis increased from 3.34% in 2002 to 8.40% in 2013. Females (84.34%) accounted for the majority of cirrhotic patients with autoimmune liver diseases. Males accounted for 80.16% of HBV cirrhosis patients and 98.02% of alcoholic cirrhosis patients.

**Conclusion:**

In Beijing 302 Hospital, the top four etiologies of cirrhosis were HBV, HCV, ALD, and autoimmune liver disease. Over the last 12 years, the prevalence of HBV cirrhosis has decreased gradually, whereas that of alcoholic cirrhosis has increased significantly.

## 1. Introduction

Recent years, viral hepatitis, especially chronic hepatitis B (CHB), is still a main reason of liver-related morbidity and mortality in China. While the prevalence of hepatitis B virus (HBV) infections fell from 10% to 7% between 1992 and 2006 as a result of a national HBV vaccination program [1]. However, due to increased national production and consumption of alcoholic beverages in China, alcoholic liver disease (ALD) is emerging as a leading cause of chronic liver disease. According to published studies, the point prevalence of ALD ranges from 2.3% to 6.1%, with a median prevalence of 4.5% in the Chinese population [2, 3]. In addition to ALD, the prevalence of autoimmune liver disease and drug-induced liver injury is increasing in China.

The aim of the present study was to investigate the variation in the disease spectrum of hospitalized patients diagnosed with liver cirrhosis in Beijing 302 Hospital between 2002 and 2013.

## 2. Study Population and Methods

### 2.1. Patients

This was a retrospective analysis of patients admitted to Beijing 302 Hospital, which is a large tertiary hospital specialized in liver diseases in Beijing. From 2002 to 2013, 82,562 patients diagnosed with liver cirrhosis were admitted to Beijing 302 Hospital. The diagnosis of cirrhosis and associated etiologies was based on clinical practice guidelines [4–9]. Patient data were obtained from the hospital's medical records. After active treatment, amelioration of symptoms, physical findings, and complications, in addition to improvements in abnormal liver function or coagulation function, was considered an improvement.

### 2.2. Statistical Analysis

Continuous variables with a normal distribution were expressed as the mean ± standard deviation (mean ± SD). Data that were not normally distributed were expressed as the median (interquartile range). An analysis of variance and SNK test were used to compare nonparametric and parametric continuous variables. Categorical variables were expressed as frequencies, with percentages. The categorical variables were analyzed by an R × C chi-square test or the Kruskal–Wallis test. Data were analyzed using SPSS version 18.0 for Windows (SPSS, Chicago, IL). Tests were two-sided, and a probability (*P*) value of less than 0.05 was considered statistically significant.

### 2.3. Ethical Approval

The study was approved by the ethics committee of Beijing 302 Hospital, and the study conformed to the Helsinki Declaration of 1977. Written informed consent was obtained from all the patients and volunteers.

## 3. Results

### 3.1. Etiologies

The etiologies of the 82,562 hospitalized cirrhotic patients are shown in Table 1. The top four etiologies of cirrhosis were HBV, HCV, ALD, and autoimmune liver diseases. Among 4080 patients with autoimmune liver cirrhosis, there were 2225 cases of autoimmune hepatitis (AIH) and 1855 cases of primary biliary cirrhosis (PBC).

### 3.2. Changes in Etiologies of Cirrhosis in the Last 12 Years

**Figure 1:**
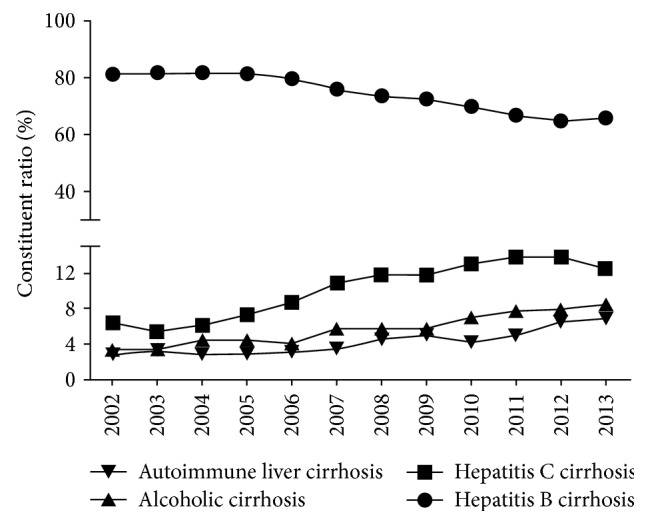
Changes in etiologies of cirrhosis from 2002 to 2013. For the hospitalized patients in Beijing 302 Hospital, the percentage of HBV cirrhosis decreased from 81.53% in 2002 to 66.00% in 2013. Cirrhosis due to HCV, ALD, and autoimmune liver disease increased gradually over time. Alcoholic cirrhosis increased 2.5 times from 3.34% in 2002 to 8.40% in 2013.

The most common etiologies of cirrhosis were HBV, HCV, ALD, and autoimmune liver disease, with a total of 77,966 patients diagnosed with these diseases in the past 12 years. Cirrhosis caused by them accounted for 94.43% of cases, as discussed in this paper. Supplemental Table 1 in Supplementary Material available online at https://doi.org/10.1155/2017/5605981 and Figure 1 show changes in the disease spectrum of cirrhosis patients in the past 12 years. The percentage of HBV cirrhosis decreased from 81.53% in 2002 to 66.00% in 2013. Cirrhosis due to HCV, ALD, and autoimmune liver disease increased gradually over time. Alcoholic cirrhosis increased 2.5 times from 3.34% in 2002 to 8.40% in 2013.

### 3.3. Gender Distribution of the Cirrhosis Patients

**Figure 2:**
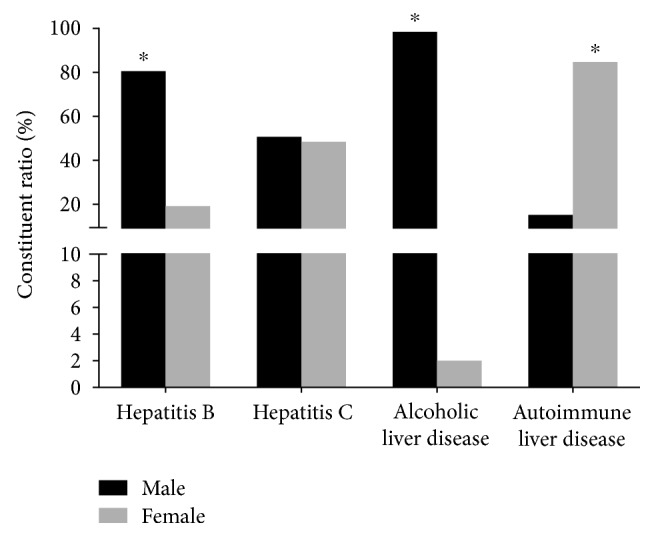
Gender distribution of the cirrhosis patients. For the inpatients in Beijing 302 Hospital, females accounted for the majority of patients with autoimmune liver cirrhosis. The percentage of female and male patients with HCV was comparative. Males accounted for the majority of HBV and alcoholic cirrhosis patients (^∗^*P* < 0.01).

Figure 2 shows the gender distribution of the cirrhosis patients. Females accounted for the majority of patients with autoimmune liver cirrhosis. The percentage of female and male patients with HCV was similar. However, males accounted for the majority of HBV and alcoholic cirrhosis patients. The gender distribution of the cirrhosis groups was significantly different (*P* < 0.01).

### 3.4. Age of the Cirrhosis Groups

The ages of the different cirrhosis groups are indicated in Table 2. Most patients with alcoholic cirrhosis and HBV were younger than 50 years, whereas most patients with HCV cirrhosis and autoimmune liver cirrhosis were older than 50 years. There was a marked difference among groups with the *P* value less than 0.01.

### 3.5. Geographic Origin of the Patients in the Different Cirrhosis Groups

**Figure 3:**
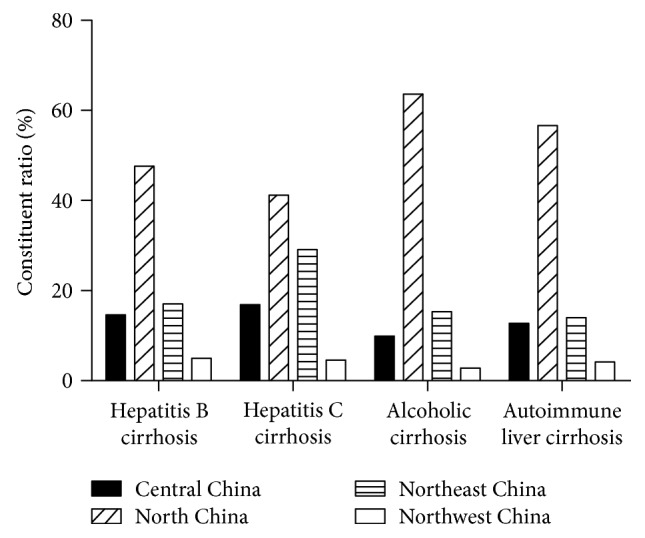
Geographic origin of the patients in the different cirrhosis groups. In this research, most patients with HBV cirrhosis, HCV cirrhosis, alcoholic cirrhosis, and autoimmune liver cirrhosis were from North China and Northeast China. North China includes Beijing, Tianjin, Hebei, Neimenggu, and Shanxi province. East China includes Shandong, Shanghai, Jiangsu, Anhui, Zhejiang, and Fujian province. Henan, Hubei, Hunan, and Jiangxi province are included in Central China. Guangdong, Guangxi, and Hainan are classified as South China. Northeast China consists of Heilongjiang, Jilin, and Liaoning. Northwest China comprises Ningxia, Qinghai, Shanxi, Xinjiang, and Gansu province. Southwest China includes Sichuan, Xizang, Yunnan, Guizhou, and Chongqing.

The geographic origins were divided into North China, East China, Central China, South China, Northeast China, Northwest China, and Southwest China.  Figure 3 and Supplemental Table 2 show the distribution and origins of the cirrhosis groups. Most cirrhotic patients came from North China. Most patients with HBV cirrhosis, HCV cirrhosis, alcoholic cirrhosis, and autoimmune liver cirrhosis were from North China and Northeast China. However, due to the selection bias in our hospital, this distribution cannot be considered to be representative of all of China.

### 3.6. Prognosis of the Patients in the Different Cirrhosis Groups

Table 3 shows the prognosis of the patients in the different cirrhosis groups. After active treatment, more than 70% of HBV and HCV cirrhosis patients and nearly 80% of alcoholic and autoimmune liver cirrhosis patients showed improvements. When compared with the other cirrhosis groups, there were significant differences (*P* < 0.01).

## 4. Discussion

Cirrhosis refers to end-stage liver disease, which is caused by multiple factors. It is associated with various complications, including ascites, upper gastrointestinal hemorrhage, hepatic encephalopathy, and spontaneous peritonitis. It is a complex disease, with a poor prognosis. Primary hepatic cancer may occur in some cirrhosis patients. Others may require a liver transplantation because of liver cancer or decompensation of liver function.

The disease spectrum of cirrhosis in China differs from that in other countries, where cirrhosis is mainly caused by ALD and hepatitis C. For example, Moreau et al. [10] reported that alcoholic cirrhosis accounted for 66.6% of all cirrhosis cases in France, whereas hepatitis C cirrhosis, alcoholic cirrhosis overlapping with viral hepatitis, and hepatitis B cirrhosis accounted for 16%, 14.7%, and 2.7% of cases, respectively. Haukeland et al. [11] reported that among 1264 patients diagnosed with cirrhosis from January 1999 to March 2004, 53% of cases were caused by ALD and the remaining cases were due to hepatitis (12%), autoimmune liver disease (12%), hemochromatosis (4%), and nonalcoholic fatty liver disease (3%). The etiology in 16% of cases was unknown. However, based on a high incidence of diabetes mellitus, the authors concluded that cirrhosis in these cases may have been caused by nonalcoholic steatohepatitis. ALD was reported to be responsible for more than 50% of cirrhosis cases in European countries. In contrast, in our research, ALD was responsible for less than 10% of cases of cirrhosis. However, as alcohol consumption continues to increase in China, the incidence of alcoholic cirrhosis will also likely increase.

There is a high incidence of HBV infection in China. According to an epidemiological investigation of hepatitis B in 2006, the carrying rate of HBsAg was 7.18% in the population from 1 year to 59 years [12]. There are around 93 million individuals with chronic HBV infection, and about 20 million of these are chronic hepatitis patients [13]. Cirrhosis is primarily the result of HBV infection. In the present study, 71.15% of the 82,562 cirrhosis cases were caused by hepatitis B. The HBV infection rate has fallen dramatically in Chinese children since the introduction of the HBV vaccine inoculation program, and the carrying rate of HBsAg today is only 0.96% among children under 5 years [12]. The incidence of hepatitis B cirrhosis also continues to decrease annually as a result of the availability of effective antiviral drugs, such as nucleoside analogs and interferon. As shown in the present study, the percentage of hepatitis B cirrhosis declined from 81.53% in 2002 to 66.00% in 2013.

Since HBV and HCV were found, several decades have passed. This explains why most of the patients with hepatitis B and C cirrhosis in the present study were middle aged to old. The incidence of HCV can be expected to decrease further as a result of strict screening of blood products and using effective antiviral drugs.

With improvements in socioeconomic conditions, alcohol consumption has increased. According to one report in China, the production of alcohol rose from 7.113 million tons in 1984 to 30.699 million tons in 2001 [14]. There have been no nationwide epidemiological investigations of ALD. However, a regional epidemiological study found that the drinking population and incidence of ALD showed an upward trend. A survey of North China reported that the ratio of intemperants increased from 0.21% in the 1980s to 14.3% in the 1990s [15]. Since the beginning of this century, in some Chinese province, the drinking population has increased from 26.98% to 43.4% and the incidence of ALD in adults has increased from 4.3% to 6.5% [2, 16, 17]. A multiple-center study indicated that from 2000 to 2004, the hospitalized ratio of ALD to all liver diseases was 2.7%, 2.9%, 3.0%, 3.6%, and 4.4%, respectively [18]. From 2002 to 2013, there were 7422 hospitalized ALD patients in Beijing 302 Hospital, with the ratio to other liver diseases rising from 1.74% in 2002 to 4.60% in 2013 [19]. This result was similar to that of a multiple-center study in China, which will be discussed in another paper [19]. In this study, the percentage of hospitalized alcoholic cirrhosis patients increased from 3.34% in 2002 to 8.40% in 2013. Alcoholic cirrhosis had become the third most common cause of cirrhosis after hepatitis B and C cirrhosis.

The results of this paper were in accord with the real situation in China (i.e., the drinking population mainly consisted of middle-aged males). In addition, the majority of patients with autoimmune liver cirrhosis were middle-aged females. In the past, due to the absence of an effective diagnostic method, autoimmune hepatitis was difficult to diagnose. However, in recent years, it has been paid more and more attention. In the present study, the majority of cirrhosis cases were patients from North China and Northeast China. However, due to selection bias in the hospital, this distribution is not representative of China.

In this study, the top four etiologies of cirrhosis in Beijing 302 Hospital were HBV, HCV, ALD, and autoimmune liver disease. Although the prevalence of hepatitis B cirrhosis has decreased, the prevalence of alcoholic cirrhosis has increased gradually. A nationwide multiple-center study is needed to detect changes in etiologies of hospitalized patients with liver cirrhosis in the whole country.

## Supplementary Material

Supplemental Table 1. The constituent ratio of different cirrhotic patients from 2002 to 2013. Supplemental table 2. The constituent ratio of native place for different cirrhotic patients.

## Figures and Tables

**Table 1:** The etiologies of 82,562 hospitalized cirrhotic patients from 2002 to 2013.

Etiology of cirrhosis	Cases	Constituent ratio (%)	Rank
Hepatitis B	58,742	71.15	1
Hepatitis C	9627	11.66	2
Alcoholic liver disease	5517	6.68	3
Autoimmune liver disease	4080	4.94	4
Cryptogenic cirrhosis	2681	3.25	5
Hepatitis B overlapping C	1119	1.36	6
Drug-induced liver injury	548	0.66	7
Hepatolenticular degeneration	128	0.16	8
Vascular obstruction disease	33	0.04	9
Nonalcoholic fatty liver disease	32	0.04	10
Bilharziasis	28	0.03	11
Cardiac cirrhosis	24	0.03	12
Malnutritional cirrhosis	3	0.00	13

**Table 2:** Age of the cirrhosis groups.

	Cases	Mean	*P* value
Hepatitis B cirrhosis	58,742	48.19 ± 11.14	0.000
Hepatitis C cirrhosis	9627	56.73 ± 10.12	
Alcoholic cirrhosis	5517	49.62 ± 10.23	
Autoimmune liver cirrhosis	4080	56.57 ± 12.21	

Compared with hepatitis B cirrhosis or alcoholic cirrhosis, *P* < 0.01.

**Table 3:** Prognosis of the patients in the different cirrhosis groups.

	Improvement	Inefficacy or death	*P* value
Hepatitis B cirrhosis	41,736 (71.05%)	17,006 (28.95%)	0.000
Hepatitis C cirrhosis	6997 (72.68%)	2630 (27.32%)	
Alcoholic cirrhosis	4365 (79.12%)	1152 (20.88%)	
Autoimmune liver cirrhosis	3261 (79.93%)	819 (20.07%)	
