# Supplementary material for: Changes in Etiologies of Hospitalized Patients with Liver Cirrhosis in Beijing 302 Hospital from 2002 to 2013
Source: Mediators Inflamm. 2017 Nov 19;2017:5605981. doi: 10.1155/2017/5605981 (PMC5735322; doi:10.1155/2017/5605981)
Supplement: Supplementary file 1 — Supplemental Table 1. The constituent ratio of different cirrhotic patients from 2002 to 2013. Supplemental table 2. The constituent ratio of native place for different cirrhotic patients. [file 5605981.f1.doc]

**Supplemental Table 1. The constituent ratio of different cirrhotic patients from 2002 to 2013**

|  | Hepatitis B cirrhosis | Hepatitis C cirrhosis | Alcoholic cirrhosis | Autoimmune cirrhosis |
| --- | --- | --- | --- | --- |
| 2002 | 81.53 | 6.39 | 3.34 | 2.77 |
| 2003 | 81.85 | 5.47 | 3.56 | 3.29 |
| 2004 | 81.73 | 6.15 | 4.45 | 2.83 |
| 2005 | 81.55 | 7.35 | 4.43 | 2.91 |
| 2006 | 79.90 | 8.71 | 4.13 | 3.08 |
| 2007 | 76.00 | 11.00 | 5.74 | 3.39 |
| 2008 | 73.62 | 11.77 | 5.77 | 4.50 |
| 2009 | 72.61 | 11.76 | 5.82 | 5.06 |
| 2010 | 70.01 | 13.14 | 7.07 | 4.19 |
| 2011 | 66.84 | 13.91 | 7.74 | 4.87 |
| 2012 | 65.00 | 13.90 | 8.00 | 6.50 |
| 2013 | 66.00 | 12.60 | 8.40 | 6.80 |

**Supplemental table 2. The constituent ratio of native place for different cirrhotic patients**

|  | Central China | North China | Northeast China | Northwest China |
| --- | --- | --- | --- | --- |
| Hepatitis B cirhosis | 8482  (14.44%) | 22469  (38.25%) | 9998  (17.02%) | 2802  (4.77%) |
| Hepatitis C cirrhosis | 1608  (16.70%) | 3129  (32.50%) | 2786  (28.94%) | 408  (4.24%) |
| Alcoholic cirrhosis | 535  (9.69%) | 2324  (42.13%) | 849  (15.39%) | 156  (2.83%) |
| Autoimmune liver cirrhosis | 515  (12.63%) | 1626  (39.86%) | 559  (13.70%) | 167  (4.09%) |
