# Supplementary material for: Changes in Etiologies of Hospitalized Patients with Liver Cirrhosis in Beijing 302 Hospital from 2002 to 2013
Source: Mediators Inflamm. 2017 Nov 19;2017:5605981. doi: 10.1155/2017/5605981 (PMC5735322; doi:10.1155/2017/5605981)
Supplement: Supplementary file 2 [file 5605981.f2.pdf]

Supplemental Table 1. The constituent ratio of different cirrhotic patients from 2002 to 2013

	Hepatitis B cirrhosis	Hepatitis C cirrhosis	Alcoholic cirrhosis	Autoimmune cirrhosis
2002	81.53	6.39	3.34	2.77
2003	81.85	5.47	3.56	3.29
2004	81.73	6.15	4.45	2.83
2005	81.55	7.35	4.43	2.91
2006	79.90	8.71	4.13	3.08
2007	76.00	11.00	5.74	3.39
2008	73.62	11.77	5.77	4.50
2009	72.61	11.76	5.82	5.06
2010	70.01	13.14	7.07	4.19
2011	66.84	13.91	7.74	4.87
2012	65.00	13.90	8.00	6.50
2013	66.00	12.60	8.40	6.80

Supplemental table 2. The constituent ratio of native place for different cirrhotic patients

	Central China	North China	Northeast China	Northwest China
Hepatitis B cirhosis	8482 (14.44%)	22469 (38.25%)	9998 (17.02%)	2802 (4.77%)
Hepatitis C cirrhosis	1608 (16.70%)	3129 (32.50%)	2786 (28.94%)	408 (4.24%)
Alcoholic cirrhosis	535 (9.69%)	2324 (42.13%)	849 (15.39%)	156 (2.83%)
Autoimmune liver cirrhosis	515 (12.63%)	1626 (39.86%)	559 (13.70%)	167 (4.09%)
